# Patient and Health Care Worker Perceptions of Communication and Ability to Identify Emotion When Wearing Standard and Transparent Masks

**DOI:** 10.1001/jamanetworkopen.2021.35386

**Published:** 2021-11-22

**Authors:** Jacqueline N. Chu, Joy E. Collins, Tina T. Chen, Peter R. Chai, Farah Dadabhoy, James D. Byrne, Adam Wentworth, Ian A. DeAndrea-Lazarus, Christopher J. Moreland, Jaime A. B. Wilson, Alicia Booth, Omkar Ghenand, Chin Hur, Giovanni Traverso

**Affiliations:** 1 Division of Gastroenterology, Massachusetts General Hospital, Harvard Medical School, Boston; 2 David H. Koch Institute for Integrative Cancer Research, Massachusetts Institute of Technology, Cambridge; 3 Division of Gastroenterology, Brigham and Women’s Hospital, Harvard Medical School, Boston, Massachusetts; 4 Massachusetts Institute of Technology, Cambridge; 5 The Fenway Institute, Fenway Health, Boston, Massachusetts; 6 Department of Emergency Medicine, Brigham and Women’s Hospital, Boston, Massachusetts; 7 Harvard Radiation Oncology Program, Boston, Massachusetts; 8 Association of Medical Professionals with Hearing Losses, Miamisburg, Ohio; 9 The University of Rochester School of Medicine and Dentistry, Rochester, New York; 10 Department of Internal Medicine, Dell Medical School at the University of Texas at Austin; 11 Department of Medicine, Columbia University Medical Center, New York, New York; 12 Department of Epidemiology, Mailman School of Public Health and Herbert Irving Comprehensive Cancer Center, Columbia University Medical Center, New York, New York; 13 Department of Mechanical Engineering, Massachusetts Institute of Technology, Cambridge

## Abstract

**Importance:**

Adoption of mask wearing in response to the COVID-19 pandemic alters daily communication.

**Objective:**

To assess communication barriers associated with mask wearing in patient-clinician interactions and individuals who are deaf and hard of hearing.

**Design, Setting, and Participants:**

This pilot cross-sectional survey study included the general population, health care workers, and health care workers who are deaf or hard of hearing in the United States. Volunteers were sampled via an opt-in survey panel and nonrandomized convenience sampling. The general population survey was conducted between January 5 and January 8, 2021. The health care worker surveys were conducted between December 3, 2020, and January 3, 2021. Respondents viewed 2 short videos of a study author wearing both a standard and transparent N95 mask and answered questions regarding mask use, communication, preference, and fit. Surveys took 15 to 20 minutes to complete.

**Main Outcomes and Measures:**

Participants’ perceptions were assessed surrounding the use of both mask types related to communication and the ability to express emotions.

**Results:**

The national survey consisted of 1000 participants (mean [SD] age, 48.7 [18.5] years; 496 [49.6%] women) with a response rate of 92.25%. The survey of general health care workers consisted of 123 participants (mean [SD] age, 49.5 [9.0] years; 84 [68.3%] women), with a response rate of 11.14%. The survey of health care workers who are deaf or hard of hearing consisted of 45 participants (mean [SD] age, 54.5 [9.0] years; 30 [66.7%] women) with a response rate of 23.95%. After viewing a video demonstrating a study author wearing a transparent N95 mask, 781 (78.1%) in the general population, 109 general health care workers (88.6%), and 38 health care workers who are deaf or hard of hearing (84.4%) were able to identify the emotion being expressed, in contrast with 201 (20.1%), 25 (20.5%), and 11 (24.4%) for the standard opaque N95 mask. In the general population, 450 (45.0%) felt positively about interacting with a health care worker wearing a transparent mask; 76 general health care workers (61.8%) and 37 health care workers who are deaf or hard of hearing (82.2%) felt positively about wearing a transparent mask to communicate with patients.

**Conclusions and Relevance:**

The findings of this study suggest that transparent masks could help improve communication during the COVID-19 pandemic, particularly for individuals who are deaf and hard of hearing.

## Key Points

**Question:**

Could transparent masks help to overcome communication barriers associated with widespread mask use among the general population, general health care workers, and health care workers who are deaf or hard of hearing in the United States?

**Findings:**

In this survey study of 1000 members of the general public, 123 general health care workers, and 45 health care workers who are deaf or hard of hearing, participants perceived mask wearing as potentially impairing communication. Respondents reported an improved ability to read emotion with transparent mask use, and transparent masks were generally accepted across all 3 populations surveyed.

**Meaning:**

These findings suggest that transparent masks have the potential to overcome barriers in communication brought on by universal mask wearing during the COVID-19 pandemic.

## Teaser

This survey study assesses communication barriers in patient-clinician interactions and among individuals who are deaf and hard of hearing when wearing standard vs transparent masks.

## Introduction

Universal mask wearing serves as a crucial public health measure in preventing the spread of COVID-19.^1^ However, studies have shown that masks may negatively affect communication and one’s ability to convey emotions such as empathy, which may impair patient-clinician relationships.^2,3^ Additionally, the use of masks presents unique challenges to individuals who are deaf or hard of hearing (DHH). Approximately 17% of all adults living in the United States experience some degree of hearing loss.^1,4,5^ Even before COVID-19, those in DHH communities already faced communication barriers in health care settings due to high noise levels and the environmental engineering of health care facilities.^6^ Medical grade masks have been found to affect sound frequency and muffle speech, by as much as 3 to 4 dB for surgical masks and 12 dB for respirators, and also prevent visualization of the lips and facial expressions.^7^ DHH health care workers (HCWs) face increasing barriers to participate in routine aspects of their work.^8^ The potential for prolonged use of face masks after the COVID-19 pandemic, especially in health care settings, heightens the importance of addressing these challenges.^9,10,11,12^

Transparent masks could help to mitigate these issues.^13^ A few transparent masks have been introduced to the market but are not widely available in most health care settings and may not meet medical grade standards.^14,15,16^ Although there has been significant focus on improving the supply of standard face masks, the widespread adoption of transparent masks remains largely unaddressed. We conducted surveys among a sample of the general population, general HCWs, and HCWs who are DHH to assess the role of widespread mask use on communication. We hypothesized that transparent mask use could improve nonverbal communication and the ability to perceive emotion through facial expressions.

## Methods

### Study Design

This observational study consisted of 3 pilot cross-sectional online surveys of 3 populations of interest: (1) a survey of opt-in panelist-members from the international survey provider, YouGov, composed of 1.8 million US residents representative of the general population; (2) a survey of general HCWs from 2 urban academic quaternary care centers in Boston, Massachusetts; and (3) a survey of DHH HCWs affiliated with the nonprofit organization Association of Medical Professionals with Hearing Losses (AMPHL). Survey questions were developed based on previously published surveys in the literature and a survey provided by the not-for-profit organization Ideas for Ears.^17^ The survey was programmed in REDCap and piloted among members of the study team to test for understanding prior to dissemination. This study followed the Strengthening the Reporting of Observational Studies in Epidemiology (STROBE) reporting guidelines for cross-sectional cohort studies.

Study review and approval was obtained from the institution review board (IRB) of Mass General Brigham (MGB). YouGov conducted the national survey of the general population between January 5 and January 8, 2021. The surveys of HCWs were conducted from December 3, 2020, to January 3, 2021. Informed consent was obtained using an IRB-approved fact sheet, after which participants were presented the survey on the YouGov platform or via REDCap. The YouGov survey was defined as a nonprobability internet panel, and the general HCW and DHH HCW surveys are defined as convenience-based nonprobability samples in accordance with the American Association for Public Opinion Research (AAPOR).^18^

National survey respondents from the general population were recruited to the opt-in panel using active volunteer sampling methods via online advertising campaigns (public surveys), permission-based email campaigns, partner-sponsored solicitations, telephone-to-web recruitment, and mail-to-web recruitment. After initial panel recruitment, participants were selected based on representativeness of the general US population. Inclusion criteria included adults older than 18 years living in the United States. Members of YouGov’s opt-in survey panels received incentive in the form of points that can be redeemed for prizes or gift cards. General and DHH HCW respondents were recruited using nonrandomized, nonprobabilistic convenience sampling via departmental email lists, with 1 follow-up reminder email sent to nonresponders approximately 2 weeks after the initial email. Inclusion criteria were HCWs older than 18 years employed through an MGB hospital and/or members of AMPHL. Participants from the HCW populations did not receive incentives or compensation. Participation in all surveys was anonymous, and all data collected were confidential. The participation rate was calculated, and raw results were tabulated. For the national sample through YouGov, weights were applied to ensure that the sample was nationally representative of the general population.

### Measurements and Covariates

Each survey lasted approximately 15 to 20 minutes (eTables 1-9 in the Supplement). Each survey included a demographic section that gathered information regarding age, sex, race, education, employment status, and household income. Race was classified by YouGov and study authors, and it was assessed to understand how views on mask wearing and mask use may vary demographically. Both surveys of HCWs consisted of additional demographic questions specific to HCWs. The DHH survey included an additional section with questions specific to hearing loss. All surveys included brief video clips showing a study author smiling while wearing a custom-designed transparent mask created by members of the study team and a standard opaque N95 mask (Figure, A).^19,20^ Respondents were then asked questions regarding whether they were able to detect the author’s emotion and whether they felt positively toward the use of transparent masks to communicate in a health care setting. They were also asked questions specific to mask wearing and communication as well as mask preference and fit in the context of the COVID-19 pandemic. All survey questions may be found in eTables 1-9 in the Supplement.

**Figure.**
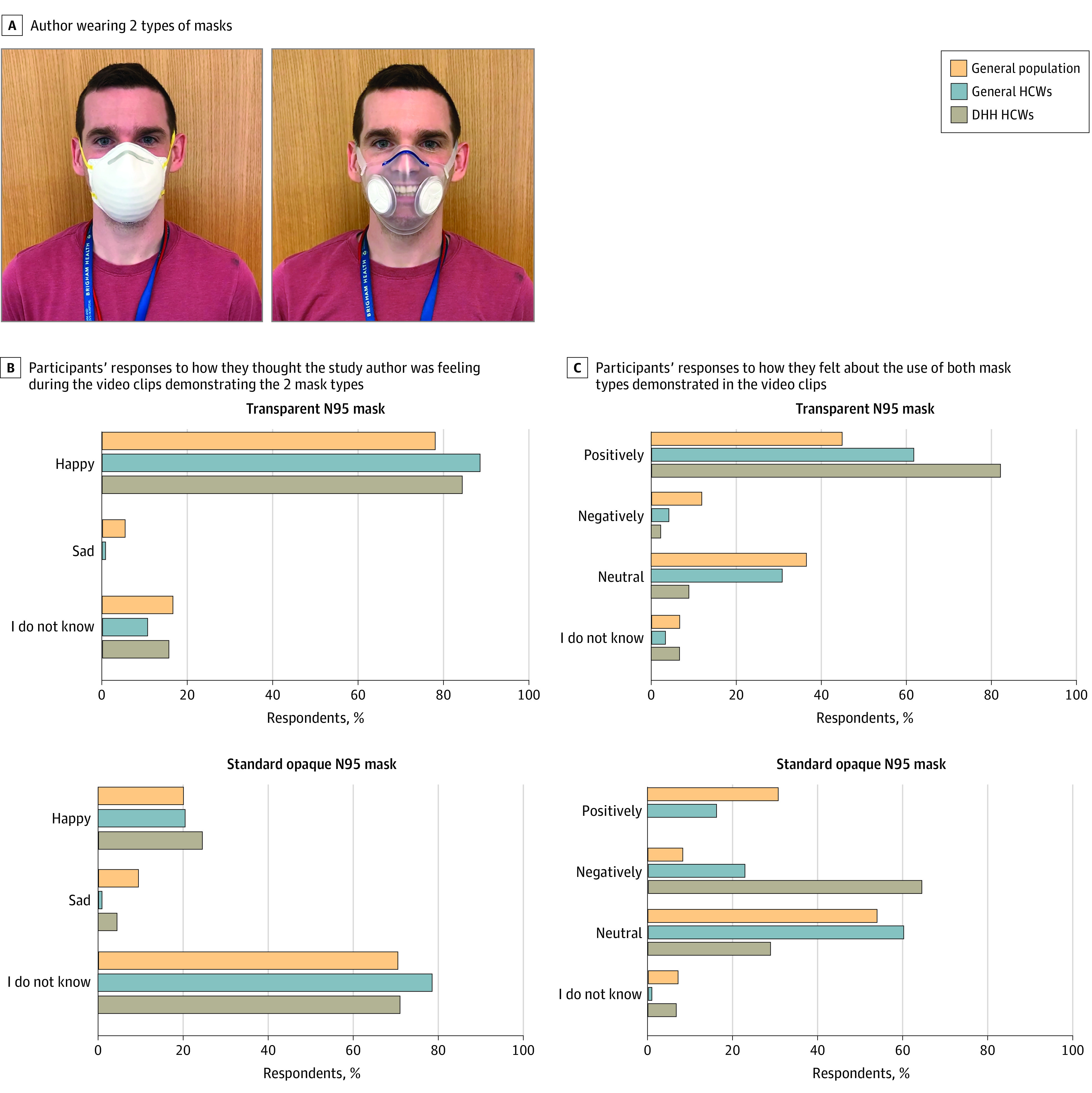
Response to Videos of a Study Author Wearing a Standard Opaque N95 Mask and a Transparent N95 Mask DHH indicates deaf or hard of hearing; HCW, health care worker.

### Statistical Analysis

Demographic data were described and presented using descriptive statistics (Stata IC version 16 [StataCorp]). To evaluate participant responses in each population to the video clips demonstrating the use of both a transparent and standard mask, a Wilcoxon matched-pairs signed-rank test was used to compare the medians of each population’s responses. Proportions of participant responses across populations for shared questions across all 3 surveys were compared using a Fisher Exact test (2-tailed test, *P* < .05 for statistical significance). To assess feedback gathered for open-ended response prompts, all responses were collected, and a framework matrix analysis was used to generate key themes that emerged from written qualitative responses (eMethods in the Supplement).^21^

## Results

### Demographic Characteristics of National General Population Survey

A total of 1279 members of YouGov’s opt-in panel were contacted to participate in the survey. Of those, 1265 participated, and 1180 completed the survey (participation rate, 92.25%). Prior to data collection, sample matching was conducted to generate a nationally representative sample of 1000 participants. The mean (SD) age of participants was 48.7 (18.5) years; 504 participants (50.4%) were men and 496 (49.6%) were women; 117 (11.7%) were Black or African American, 140 (14.0%) Hispanic, and 671 (67.1%) White; a total of 657 (65.7%) had received education beyond high school (Table 1).

**Table 1.** Respondent Demographic Characteristics

Characteristic	Respondents, No. (%)			
	General population (n = 1000)	General HCWs (n = 123)	DHH HCWs (n = 45)	Total (N = 1168)
Age, y				
18-24	111 (11.10)	5 (4.07)	1 (2.22)	117 (10.02)
25-34	180 (18.00)	41 (33.33)	10 (22.22)	231 (19.78)
35-44	160 (16.00)	32 (26.02)	7 (15.56)	199 (17.04)
45-54	115 (11.50)	22 (17.89)	11 (24.44)	148 (12.67)
55-64	195 (19.50)	16 (13.01)	12 (26.67)	22 (19.09)
65-74	152 (15.20)	5 (4.07)	4 (8.89)	161 (13.78)
≥75	86 (8.60)	2 (1.63)	0	89 (7.62)
Gender				
Male	504 (50.40)	39 (31.71)	15 (33.33)	558 (47.77)
Female	496 (49.60)	84 (68.29)	30 (66.67)	610 (52.23)
Race				
White	671 (67.10)	90 (73.17)	41 (91.11)	802 (68.66)
Black or African American	117 (11.70)	3 (2.44)	1 (2.22)	121 (10.36)
Hispanic or Latino	140 (14.0)	3 (2.44)	2 (4.44)	140 (11.99)
Asian or Asian American	23 (2.30)	17 (13.82)	1 (2.22)	41 (3.51)
Native American	0	0	0	0
Native Hawaiian or other Pacific Islander	11 (1.10)	0	0	11 (0.94)
Middle Eastern	2 (0.20)	0	0	2 (0.17)
South Asian	0	5 (4.07)	2 (4.44)	7 (0.60)
Multiracial	21 (2.10)	2 (1.63)	0	23 (1.97)
Other	15 (1.50)	3 (2.44)	0	18 (1.54)
Education				
Did not graduate from high school	45 (4.50)	0	0	45 (3.85)
High school graduate	298 (29.80)	1 (0.81)	1 (2.22)	300 (25.68)
Some college, but no degree (yet)	172 (17.20)	1 (0.81)	0	173 (14.81)
2-y college degree	161(16.10)	6 (4.88)	0	167 (14.30)
4-y college degree	203 (20.30)	32 (26.02)	9 (20.0)	244 (20.89)
Postgraduate degree	121 (12.10)	81 (65.85)	35 (77.78)	237 (20.29)
Unanswered	0	2 (1.63)	0	2 (0.17)
Employment				
Working full time now	284 (28.40)	110 (89.43)	27 (60.00)	421 (36.04)
Working part time now	90 (9.00)	10 (8.13)	10 (22.22)	110 (9.42)
Temporarily laid off	28 (2.80)	0	0	28 (2.40)
Unemployed	109 (10.90)	0	3 (6.67)	112 (9.59)
Retired	261 (26.10)	1 (0.81)	1 (2.22)	263 (22.52)
Permanently disabled	68 (6.80)	0	1 (2.22)	69 (5.91)
Taking care of home or family	75 (7.50)	0	0	76 (6.32)
Student	73 (7.30)	0	0	73 (6.25)
Other	12 (1.20)	1 (0.81)	3 (6.67)	16 (1.41)
Unanswered	0	1 (0.81)	0	1 (0.09)
Income, $				
<10 000	68 (6.80)	0	1 (2.22)	69 (5.91)
10 000-49 999	368 (36.80)	9 (7.32)	5 (11.11)	382 (32.71)
50 000-99 999	284 (28.40)	27 (21.95)	17 (37.78)	328 (28.08)
100 000-149 999	98 (9.80)	16 (13.01)	9 (20.00)	123 (10.53)
≥150 000	66 (6.60)	68 (55.28)	13 (28.89)	147 (12.59)
Prefer not to say	116 (11.60)	0	0	116 (9.93)
Unanswered	0	3 (2.44)	0	3 (0.26)
Are you currently wearing a mask to see patients?/Are you currently wearing a mask out in public?				
Yes, I always wear a mask	774 (77.40)	119 (96.75)	39 (86.67)	932 (79.79)
Yes, I sometimes wear a mask	173 (17.30)	2 (1.63)	3 (6.67)	178 (15.24)
No, I do not wear a mask	53 (5.30)	2 (1.63)	3 (6.67)	58 (4.97)

Abbreviations: DHH, deaf or hard of hearing; HCW, health care worker.

### Demographic Characteristics of Survey of General Healthcare Workers

A total of 1104 individuals were contacted to participate via MGB’s departmental email lists. Of those who received a recruitment email, 177 participated, and 123 completed the online survey (participation rate, 11.14%). The mean (SD) age of participants was 49.5 (9.0) years; 39 participants (31.7%) were men, and 84 (68.3%) were women; 3 (2.4%) were Black, 3 (2.4%) Hispanic, and 90 (73.2%) White; and a total of 81 (65.9%) had received a postgraduate degree (Table 1). Respondents’ answers to questions regarding health care occupation and mask use are described in eTable 4 in the Supplement.

### Survey of DHH Healthcare Workers

#### Demographic Characteristics

A total of 196 individuals were contacted to participate via AMPHL’s email lists. Of those, 66 participated in the online survey, and 45 completed the survey (participation rate, 23.95%). The mean (SD) age of participants was 54.5 (9.0) years; 15 participants (33.3%) were men, and 30 (66.7%) were women; 1 (2.2%) was Black, 2 (4.4%) were Hispanic, and 41 (91.1%) were White; a total of 35 (77.8%) had received a postgraduate degree. Respondents’ answers to questions regarding health care occupation and mask use are described in eTable 4 in the Supplement.

#### Questions Regarding Hearing Loss

Overall, 31 DHH HCW respondents (68.9%) reported a moderately severe to profound hearing loss; 35 (77.8%) and 31 (68.9%) reported preferred methods of communication as auditory input or listening and speechreading, respectively. A total of 38 DHH HCW respondents (84.4%) used an assistive listening device (ALD) for hearing assistance, and 27 (60.0%) reported experiencing mask-fit interference with their ALD. Overall, 32 DHH HCW respondents (71.1%) reported concern that face masks would make living with hearing loss much more difficult. These responses are described in further detail in eTable 8 in the Supplement.

### All Populations

#### Communication

After viewing the video clip of a study author smiling while wearing a standard opaque N95 mask, 201 respondents (20.1%), 25 respondents (20.5%), and 11 respondents (24.4%) in the general, HCW, and DHH HCW populations, respectively, were able to identify the emotion being expressed (237 [20.3%] across all populations). In contrast, after viewing the video of the study author wearing a transparent mask, 781 respondents (78.1%), 109 respondents (88.6%), 38 respondents (84.4%) in the general, HCW, and DHH HCW populations, respectively, were able to identify the emotion being expressed (928 [79.5%] across all populations; *P* < .001) (Figure, B and eTable 9 in the Supplement). Overall, 450 respondents in the general population (45.0%) felt positively and 366 (36.6%) felt neutrally about interacting with an HCW who was wearing a transparent mask; 76 general HCWs (61.8%) and 37 DHH HCWs (82.2%) felt positively about wearing a transparent mask to communicate with patients (*P* < .001) (Figure, C and eTable 9 in the Supplement).

In the general population, 518 respondents (51.8%) reported having trouble communicating while wearing a face mask since the COVID-19 pandemic. In the HCW populations, 92 general HCWs (74.8%) and 44 DHH HCWs (97.8%) had trouble communicating while wearing a face mask (*P* < .001), with 36 DHH HCWs (80.0%) reporting difficulty at a moderate to high level (Table 2). In addition, 416 respondents from the general population (41.6%), 84 general HCWs (69.0%), and 39 DHH HCWs (86.7%) felt communication with others would be more difficult while wearing a standard mask in contrast to a transparent mask (*P* < .001). Overall, 379 respondents from the general population (37.9%), 75 general HCWs (61.0%), and 41 DHH HCWs (91.1%) felt it would be easier to understand or hear people who wore a transparent mask (*P* < .001) (Table 2). Additionally, 339 respondents from the general population (33.9%) felt they would be more at ease seeing a HCW who wore a transparent mask, while 75 general HCWs (61.0%) and 34 DHH HCWs (75.6%) felt that patients would be more at ease with a HCW who wore a transparent mask (Table 2). Overall, 302 respondents from the general population (30.2%), 65 general HCWs (52.9%), and 41 DHH HCWs (91.1%) preferred the use of transparent masks over standard masks (*P* < .001) (Table 2).

**Table 2.** The Perceived Impact of Mask Wearing on Communication

Question	Respondents, No. (%)				
	General population (n = 1000)	General HCWs (n = 123)	DHH HCWs (n = 45)	Total (N = 1168)^a^	*P* value^b^
Do you generally find it harder to communicate with people/patients who are wearing standard masks?					
Harder to communicate	416 (41.60)	84 (68.85)	39 (86.67)	539 (46.19)	<.001
Neutral, equally well	478 (47.80)	26 (21.31)	1 (2.22)	505 (43.19)	
I haven’t spoken to anyone wearing a mask	24 (2.40)	NA	5 (11.11)	29 (2.49)	
I do not know	82 (8.02)	12 (9.84)	0	94 (8.05)	
Do you think you would be able to understand or hear people/patients better if they wore a transparent mask?					
Yes, easier to communicate	379 (37.90)	75 (60.98)	41 (91.11)	495 (42.38)	<.001
Neutral, equally well	440 (44.00)	26 (21.14)	1 (2.22)	467 (39.98)	
I haven’t spoken to any people/patients wearing a mask	30 (3.00)	2 (1.63)	NA	32 (2.74)	
I do not know	151 (15.10)	20 (16.26)	3 (6.67)	174 (14.90)	
Do you think patients would feel more or less at ease if the clinician was wearing a transparent mask?					
More at ease	NA	75 (60.98)	34 (75.56)	109 (64.88)	NA
Less at ease	NA	6 (4.88)	2 (4.44)	8 (4.76)	
No difference	NA	17 (13.82)	0	17 (10.12)	
I do not know	NA	25 (20.33)	9 (20.00)	34 (20.24)	
Do you think you would feel more or less at ease if the clinician was wearing a transparent mask?					
More at ease	339 (33.90)	NA	NA	339 (33.90)	NA
Less at ease	201 (20.10)	NA	NA	201 (20.10)	
No difference	422 (42.20)	NA	NA	422 (42.20)	
I do not know	38 (3.80)	NA	NA	38 (3.80)	
Have you experienced any difficulty communicating with patients/others who are wearing face masks?					
Yes	518 (51.8)	92 (74.80)	44 (97.78)	654 (55.99)	<.001
No	482 (48.2)	31 (25.20)	1 (2.22)	514 (44.01)	
If yes to question above, how would you rate the level of difficulty?					
Low, some difficulty	221 (22.10)	60 (48.78)	6 (13.33)	287 (24.57)	NA
Moderate, considerable difficulty	203 (20.30)	27 (21.95)	18 (40.00)	248 (21.23	
High, difficult	71 (7.10)	4 (3.25)	18 (40.00)	93 (7.96)	
Not difficult	19 (1.90)	NA	NA	19 (1.63)	
Other	4 (0.40)	0	2 (4.44)	6 (0.51)	
NA	NA	NA	1 (2.22)	1 (0.09)	
What challenges have the use of face masks created for you when communicating with others? Please select all that apply					
None	222 (22.20)	14 (11.38)	1 (2.22)	237 (20.29)	NA
Prevented ability to lip read	296 (29.60)	54 (43.90)	41 (91.11)	391 (33.48)	
Muffled voices/reduced sound clarity	579 (57.90)	97 (78.86)	40 (88.89)	716 (61.30)	
Reduced volume/sound of person’s voice	437 (43.70)	86 (69.92)	29 (64.44)	552 (47.26)	
I do not know	56 (5.60)	1 (0.81)	1 (2.22)	58 (4.97)	
Other	40 (4.00)	8 (6.50)	2 (4.44)	50 (4.28)	
What communication challenges are you concerned about regarding the use of face masks? Please select all that apply					
None	261 (26.10)	13 (10.57)	1 (2.22)	275 (23.54)	NA
Prevented ability to lip read	235 (23.50)	51 (41.46)	40 (88.89)	326 (27.91)	
Muffled voiced/reduced sound clarity	529 (52.90)	87 (70.73)	38 (84.44)	654 (55.99)	
Reduced volume/sound of person’s voice	410 (41.00)	82 (66.67)	32 (71.11)	524 (44.86)	
Misunderstanding/not hearing others	420 (42.00)	87 (70.73)	35 (77.78)	542 (46.40)	
I do not know	48 (4.80)	3 (2.44)	0	51 (4.37)	
Other	19 (1.90)	6 (4.88)	1 (2.22)	26 (2.23)	
Overall, did you prefer the use of 1 of the masks (transparent vs standard) over the other?					
Transparent mask	302 (30.20)	65 (52.85)	41 (91.11)	408 (34.93)	<.001
Standard mask	267 (26.70)	10 (8.13)	0	277 (23.72)	
No preference	394 (39.40)	31 (25.20)	3 (6.67)	428 (36.64)	
I do not know	37 (3.70)	17 (13.82)	1 (2.22)	55 (4.71)	

Abbreviations: DHH, deaf or hard of hearing; HCW, health care worker; NA, not applicable.

^a^ Questions that were not asked of specific groups are labeled NA.

^b^ P < .05 was considered statistically significant.

Respondents were asked about specific communication challenges the use of face masks had created for them as well as population-specific questions surrounding clinician and patient communication. Respondent rates of specific challenges and thoughts about mask wearing and clinician and patient communication are found in Table 2 and Table 3.

**Table 3.** Clinician and Patient Communication

Question	Respondents, No. (%)
General Population, 1000 respondents	
Considering your relationship with your own clinician(s), do you think that your comfort level speaking with them would be different based on what type of mask they were using?	
More comfortable if they were wearing a transparent mask	247 (24.70)
More comfortable if they were wearing a standard mask	168 (16.80)
No preference	470 (47.00)
I do not have a clinician	47 (4.70)
I do not know	68 (6.80)
Would you prefer to see a clinician who was wearing a transparent mask or one who was wearing a standard mask?	
A clinician who wore a transparent mask	280 (28.00)
A clinician who wore a standard mask	179 (17.90)
No preference	489 (48.90)
I do not know	52 (5.20)
Which health care clinician would you trust more to care for you?	
A clinician who wore a transparent mask	157 (15.70)
A clinician who wore a standard mask	153 (15.30)
No preference	629 (62.90)
I do not know	61 (6.10)
How do you feel about wearing masks as a public health response to COVID-19?	
I agree with wearing masks as a public health measure	701 (70.10)
I neither agree nor disagree with wearing masks as a public health measure	137 (13.70)
I disagree with wearing masks as a public health measure	112 (11.20)
I do not know	50 (5.00)
**General HCWs, 123 respondents**	
Do you feel that you would be able to communicate more effectively/convey empathy better with patients if you were wearing a transparent mask?	
I feel I would be able to communicate better while wearing transparent mask	95 (77.24)
Equally well, whether I am wearing a transparent or standard mask	22 (17.89)
I do not know	6 (4.88)
**DHH HCWs, 45 respondents**	
Do you feel that you would be able to communicate more effectively/convey empathy better with patients if you were wearing a transparent mask?	
Communicate better while wearing a transparent mask	41 (91.11)
Equally well, whether I am wearing a transparent or standard mask	2 (4.44)
I do not know	2 (4.44)
Did you think patients would feel more at ease if the clinician was wearing a transparent mask?	
More at ease if clinicians wore a transparent mask	34 (75.56)
Less at ease if clinicians wore a transparent mask	2 (4.44)
No difference	0
I do not know	9 (20.00)

Abbreviations: DHH, deaf or hard of hearing; HCW, health care worker.

Survey respondents provided feedback regarding challenges and concerns surrounding mask wearing in their daily life in a prompt that asked participants to provide open-ended feedback (eMethods in the Supplement). We discovered 3 important themes in responses: communication, physical discomfort, and effect on work. There was concern in all populations surrounding the loss of facial and other nonverbal cues. General HCWs commented on being unable to interpret a patient’s mood or state of mind and were also concerned about not being heard by patients. DHH HCWs commented on the difficulty of having to communicate that they were DHH to others. Regarding physical discomfort, several in the general population reported concern regarding the fogging up of glasses. General HCWs also noted discomfort from prolonged mask-wearing. Regarding mask wearing and difficulties presented at work, DHH HCWs were most notably affected. Several DHH HCWs reported that they had to switch to telehealth. One respondent reported being laid off/furloughed, while another said they had to retire prematurely. In addition, others said they were limited to working in certain environments where transparent masks were available. Those who remained working in person reported difficulty performing their job and stress and fear of missed communication with their colleagues and patients (eTable 10 in the Supplement).

#### Mask Type and Preference

Respondents were asked a series of questions concerning mask type, preference, and fit. Most respondents in all 3 populations had not used a transparent mask in the last month. Preferences in physical features as well as acceptance of incorporation of advanced technologies into masks varied significantly between populations and are further described in eTable 11 in the Supplement.

## Discussion

Our study found that there is a need to address communication barriers related to mask use, especially among people who are DHH. Furthermore, we found that the use of transparent masks is generally accepted and could help to improve communication in both public and health care settings.

Prior studies performed before COVID-19 have shown that transparent masks improve speech understanding in DHH and normal-hearing populations and that patients perceive greater empathy and trust from surgeons communicating with transparent masks.^13,22^ Our study expands on these findings by surveying HCWs in addition to the general public and characterizing the challenges faced by HCWs, especially DHH HCWs, with masks in patient care. Unlike studies performed before the pandemic, the populations surveyed in our study have had considerable personal experience with wearing face masks, and the challenges identified by respondents will help to inform future mask designs.^2,3,7,22^ In all 3 survey populations, respondents were more likely to perceive the study author’s smile with a transparent mask compared with a standard mask. In addition, the use of transparent masks was viewed as acceptable, viewed positively, and accepted by a plurality of all survey populations. These findings suggest that transparent masks are acceptable among the broader population and may be an alternative that can be integrated into available mask forms for both the general population setting and in health care settings. However, factors such as discomfort, fogging of the transparent window, and increased reduction in sound quality may be barriers to implementation of transparent masks and should be considered in transparent mask designs.^22,23^

Of all populations surveyed, we found that DHH HCWs seemed to experience more difficulties associated with the widespread use of standard masks in everyday life and health care settings at work. DHH HCWs have had to drastically change the way that they work during the pandemic.^8,24^ Although this study did not measure other dimensions of communication such as speech interpretation and other grammatical features key in American Sign Language, we believe that our survey findings suggest that these may also be affected by mask use. Further research is necessary to measure this. In addition, some DHH HCWs expressed fear of isolation and lack of independence because of widespread face mask use. Our study did not look at the association between mental health and the use of face masks; however, our findings indicate that further research in this area is needed as well as policies to ensure that communities of people who have a disability are having their communication needs met.

We found that general HCWs agreed that transparent masks would be helpful to them as well, though to a lesser degree. However, general HCWs also have contact with DHH patients. Approximately 72% of people older than 65 years experience hearing loss, and individuals in this age group have higher hospitalization rates.^25^ This is an important additional population that would benefit from increased availability of transparent masks in health care.

### Limitations

This study has limitations. This was a pilot study using a self-designed survey that has not previously been validated. Further repetitions of this study are needed to test the reliability of our findings. Follow-up studies should also include demonstration of a wider range of emotions beyond a smile. The sample sizes of both general HCW and DHH HCW populations were small and limited to 1 health care system and 1 DHH organization, respectively, which may not be representative of HCWs in the United States as a whole. Further sampling of HCWs nationally, including those who are DHH, is needed. Additionally, a nonrandomized sampling technique was used for both the general HCW and DHH HCW populations; therefore, volunteer, selection, and nonresponse bias were not accounted for. For the general population survey through YouGov, previously validated techniques, such as sample matching and weight adjustment, were used; however, substantial bias can occur with internet-based nonprobabilistic opt-in panels, such as the need for access to the internet and opt-in panel membership. In addition, the national survey was conducted through a national sampling platform of US residents, and respondents’ beliefs and attitudes surrounding mask use may vary depending on their personal experiences. Additionally, we did not assess the representation of DHH people in our survey of the general population. A survey that specifically looks at deafness and hearing loss in the general population would provide further insight into the needs of DHH communities outside of health care settings, including the inherent variety in their communication modalities. Additionally, this study did not evaluate the costs of transparent masks, which could limit their availability.^19^ However, we have conducted a prior study comparing costs of multiple respirator-use strategies, including distribution of 1 reusable, transparent respirator to all US health care workers, and found this to be less costly than the current practice of using disposable standard respirators.^14,15,16,19^ Further cost-benefit studies of transparent masks should be performed as they become more commercially available.

## Conclusions

The findings of this study suggest that widespread mask use impairs nonverbal communication and the ability to convey emotions. Transparent masks have the potential to alleviate stressors surrounding communication introduced by widespread standard mask wearing. This pilot study provides further support for transparent masks’ utility in supporting nonverbal communication, especially for those who are DHH. Our study suggests that transparent masks are needed by DHH HCWs and are considered acceptable in the general population and general health care settings, suggesting feasibility of implementation.

## Notes

Supplement. **eTable 1. **General Population Demographic Characteristics **eTable 2. **General Population Survey Questions **eTable 3. **General Health Care Worker Demographic Characteristics **eTable 4. **Questions Regarding Health Care Occupation and Mask Use at Work **eTable 5. **General Health Care Worker Survey Questions **eTable 6. **Demographic Characteristics for DHH Health Care Workers **eTable 7. **Questions Related to Hearing Loss **eTable 8. **DHH Health Care Worker Survey Questions **eTable 9. **Response to Videos of a Study Author Wearing a Transparent N95 Mask and Standard Opaque N95 Mask
eMethods.
**eTable 10. **Subset of Respondents’ Comments from Each Population on Challenges and Concerns Regarding the Widespread Use of Facemasks **eTable 11. **Mask Preference and Type

## References

[1] Noguchi Y. Demand surges for see-through face masks as pandemic swells. NPR. July 28, 2020. Accessed October 21, 2021. https://www.npr.org/sections/health-shots/2020/07/28/893071631/demand-surges-for-see-through-face-masks-as-pandemic-swells

[2] Wong CKM, Yip BHK, Mercer S, . Effect of facemasks on empathy and relational continuity: a randomised controlled trial in primary care. BMC Fam Pract. 2013;14(1):200-200. doi:10.1186/1471-2296-14-200 24364989 PMC3879648

[3] Tang JI, Shakespeare TP, Zhang XJ, . Patient satisfaction with doctor-patient interaction in a radiotherapy centre during the severe acute respiratory syndrome outbreak. Australas Radiol. 2005;49(4):304-311. doi:10.1111/j.1440-1673.2005.01467.x 16026437 PMC7185414

[4] McKee M, Moran C, Zazove P. Overcoming additional barriers to care for deaf and hard of hearing patients during COVID-19. JAMA Otolaryngol Head Neck Surg. 2020;146(9):781-782. doi:10.1001/jamaoto.2020.1705 32692807

[5] Moreland CJ, Ruffin CV, Morris MA, McKee M. Unmasked: how the COVID-19 pandemic exacerbates disparities for people with communication-based disabilities. J Hosp Med. 2021;16(3):185-188. doi:10.12788/jhm.3562 33617440

[6] Chodosh J, Weinstein BE, Blustein J. Face masks can be devastating for people with hearing loss. BMJ. 2020;370:m2683-m2683. doi:10.1136/bmj.m2683 32646862

[7] Marler H, Ditton A. “I’m smiling back at you”: exploring the impact of mask wearing on communication in healthcare. Int J Lang Commun Disord. 2021;56(1):205-214. doi:10.1111/1460-6984.12578 33038046 PMC7675237

[8] Grote H, Izagaren F. COVID-19: the communication needs of D/deaf healthcare workers and patients are being forgotten. BMJ. 2020;369:m2372. doi:10.1136/bmj.m2372 32540861

[9] Farr C. Will masks become the ‘new normal’ even after the pandemic has passed? some Americans say so. CNBC. October 24, 2020. Accessed October 22, 2021. https://www.cnbc.com/2020/10/24/will-masks-become-the-new-normal-even-after-the-pandemic-has-passed-some-americans-say-so-.html

[10] Kortepeter M. Why you’ll still need to wear a mask even after COVID-19 vaccines arrive. Forbes. October 20, 2020. Accessed October 22, 2021. https://www.forbes.com/sites/coronavirusfrontlines/2020/10/20/why-youll-still-need-to-wear-a-mask-even-after-covid-19-vaccines-arrive/?sh=600022ab5a42

[11] Olsen SJ, Azziz-Baumgartner E, Budd AP, . Decreased influenza activity during the COVID-19 pandemic—United States, Australia, Chile, and South Africa, 2020. MMWR Morb Mortal Wkly Rep. 2020;69(37):1305-1309. doi:10.15585/mmwr.mm6937a6 32941415 PMC7498167

[12] BBC News. COVID: masks and social distancing ‘could last years.’ March 21, 2021. Accessed October 22, 2021. https://www.bbc.com/news/uk-56475807

[13] Atcherson SR, Mendel LL, Baltimore WJ, . The effect of conventional and transparent surgical masks on speech understanding in individuals with and without hearing loss. J Am Acad Audiol. 2017;28(1):58-67. doi:10.3766/jaaa.15151 28054912

[14] Jelli M1. Accessed October 21, 2021. https://jellim.com/

[15] ClearMaskTM. See the person, not the mask. Accessed October 21, 2021. https://www.theclearmask.com/

[16] BendShapeTM. Highest filtration clear mask. Accessed October 21, 2021. https://bendshapemask.com/

[17] Ideas for Ears. Accessed October 21, 2021. https://www.ideasforears.org.uk/

[18] American Association for Public Opinion Research. *Final Dispositions of Case Codes and Outcomes Rates for Surveys*. 9th ed. 2016. Accessed October 25, 2021. https://www.aapor.org/AAPOR_Main/media/publications/Standard-Definitions20169theditionfinal.pdf

[19] Chu J, Ghenand O, Collins J, . Thinking green: modelling respirator reuse strategies to reduce cost and waste. BMJ Open. 2021;11(7):e048687. doi:10.1136/bmjopen-2021-048687 34275864 PMC8290946

[20] Wentworth AJ, Byrne JD, Orguc S, . Prospective evaluation of the transparent, elastomeric, adaptable, long-lasting (TEAL) respirator. ACS Pharmacol Transl Sci. 2020;3(6):1076-1082. doi:10.1021/acsptsci.0c00157 33330837 PMC7671102

[21] Guest G, MacQueen KM, Namey EE. Applied Thematic Analysis. SAGE. 2011. Accessed October 22, 2021. https://us.sagepub.com/en-us/nam/applied-thematic-analysis/book233379

[22] Kratzke IM, Rosenbaum ME, Cox C, Ollila DW, Kapadia MR. Effect of clear vs standard covered masks on communication with patients during surgical clinic encounters: a randomized clinical trial. JAMA Surg. 2021;156(4):372-378. doi:10.1001/jamasurg.2021.0836 33704389 PMC7953334

[23] McDowell, R, Watson, C, Finley, E. More speech degradations and considerations in the search for transparent face coverings during the COVID-19 pandemic. Audiology Today. Accessed October 22, 2021. https://www.audiology.org/news-and-publications/audiology-today/articles/more-speech-degradations-and-considerations-in-the-search-for-transparent-face-coverings-during-the-covid-19-pandemic/

[24] Mahase, E. COVID-19: D/deaf healthcare workers faced “widespread, systemic discrimination” during pandemic, study finds. May 26, 2021. Accessed October 22, 2021. https://www-bmj-com.ezproxymcp.flo.org/content/373/bmj.n1365 10.1136/bmj.n1365 34039616

[25] West JS, Franck KH, Welling DB. Providing health care to patients with hearing loss during COVID-19 and physical distancing. Laryngoscope Investig Otolaryngol. 2020;5(3):396-398. doi:10.1002/lio2.382 32587888 PMC7262136

