# Supplementary material for: Patient and Health Care Worker Perceptions of Communication and Ability to Identify Emotion When Wearing Standard and Transparent Masks
Source: JAMA Netw Open. 2021 Nov 22;4(11):e2135386. doi: 10.1001/jamanetworkopen.2021.35386 (PMC8609412; doi:10.1001/jamanetworkopen.2021.35386)
Supplement: Supplement. — eTable 1. General Population Demographic Characteristics eTable 2. General Population Survey Questions eTable 3. General Health Care Worker Demographic Characteristics eTable 4. Questions Regarding Health Care Occupation and Mask Use at Work eTable 5. General Health Care Worker Survey Questions eTable 6. Demographic Characteristics for DHH Health Care Workers eTable 7. Questions Related to Hearing Loss eTable 8. DHH Health Care Worker Survey Questions eTable 9. Response to Videos of a Study Author Wearing a Transparent N95 Mask and Standard Opaque N95 Mask eMethods. eTable 10. Subset of Respondents’ Comments from Each Population on Challenges and Concerns Regarding the Widespread Use of Facemasks eTable 11. Mask Preference and Type [file jamanetwopen-e2135386-s001.pdf]

Supplemental Online Content

Chu JN, Collins JE, Chen TT, et al. Patient and health care worker perceptions of communication and ability to identify emotion when wearing standard and transparent masks. *JAMA Netw Open.* 2021;4(11):e2135386. doi:10.1001/jamanetworkopen.2021.35386

eTable 1. General Population Demographic Characteristics

eTable 2. General Population Survey Questions

eTable 3. General Health Care Worker Demographic Characteristics

eTable 4. Questions Regarding Health Care Occupation and Mask Use at Work

eTable 5. General Health Care Worker Survey Questions

eTable 6. Demographic Characteristics for DHH Health Care Workers

eTable 7. Questions Related to Hearing Loss

eTable 8. DHH Health Care Worker Survey Questions

eTable 9. Response to Videos of a Study Author Wearing a Transparent N95 Mask and Standard Opaque N95 Mask

eMethods.

eTable 10. Subset of Respondents' Comments from Each Population on Challenges and Concerns Regarding the Widespread Use of Facemasks

eTable 11. Mask Preference and Type

This supplemental material has been provided by the authors to give readers additional information about their work.

© 2021 Chu JN et al. *JAMA Network Open.*

eTable 1. General Population Demographic Characteristics

Survey Question	n (%)
**Age**	
18-24 years old	111 (11.10)
25-34 years old	181 (18.10)
35-44 years old	160 (16.00)
45-54 years old	115 (11.50)
55-64 years old	195 (19.50)
65-74 years old	152 (15.20)
Over 75 years old	86 (8.60)
**Gender**	
Male	504 (50.40)
Female	496 (49.60)
**Race**	
White	671 (67.10)
Black or African American	117 (11.70)
Hispanic or Latino	140 (14.0)
Asian or Asian-American	23 (2.30)
Native American	0 (0.00)
Native Hawaiian or Other Pacific Islander	11 (1.10)
Middle Eastern	2 (0.20)
South Asian	0 (0.00)
Mixed Race	21 (2.10)
Other	15 (1.50)
**Education**	
Did not graduate from high school	45 (4.50)
High school graduate	298 (29.80)
Some college, but no degree (yet)	172 (17.20)
2-year college degree	161(16.10)
4-year college degree	203 (20.30)
Postgraduate degree	121 (12.10)
Unanswered	0 (0.00)
**Employment**	
Working full time now	284 (28.40)
Working part time now	90 (9.00)
Temporarily laid off	28 (2.80)
Unemployed	109 (10.90)
Retired	261 (26.10)
Permanently disabled	68 (6.80)
Taking care of home or family	75 (7.50)
Student	73 (7.30)
Other	12 (1.20)
Unanswered	0 (0.00)
**Income**	
Less than $10,000	68 (6.80)
$10,000 - $50,000	368 (36.80)
$50,000-$100,000	284 (28.40)
$100,000-$150,000	98 (9.80)

© 2021 Chu JN et al. *JAMA Network Open.*

Greater than $150,000	66 (6.60)
Prefer not to say	116 (11.60)
Unanswered	0 (0.00)
**Are you currently wearing a mask out in public?**	
Yes, I always wear a mask	774 (77.40)
Yes, I sometimes wear a mask	173 (17.30)
No, I do not wear a mask	53 (5.30)
**n (Total) = 1000**	

© 2021 Chu JN et al. *JAMA Network Open.*

eTable 2. General Population Survey Questions

Survey Question	n (%)
**Considering the video you have just watched (video #1), how was the healthcare provider feeling?**	
Happy	781 (78.10)
Sad	53 (5.30)
I do not know	166 (16.60)
**Consider the video you just watched (video #1) demonstrating the use of a transparent mask. How would you feel if a healthcare provider you are interacting with wore a transparent mask?**	
Positively	450 (45.00)
Negatively	118 (11.80)
Neutral	366 (36.60)
I do not know	66 (6.60)
**Considering the video you have just watched (video #2), how was the healthcare provider feeling?**	
Happy	201 (20.10)
Sad	94 (9.40)
I do not know	705 (70.50)
**Consider the video you just watched (video #2) demonstrating the use of a standard mask. How would you feel if a healthcare provider you are interacting with wore a standard mask?**	
Positively	308 (30.80)
Negatively	82 (8.20)
Neutral	540 (54.00)
I do not know	70 (7.00)
**Did you feel more or less at ease when the doctor was wearing a transparent vs. a standard mask?**	
More at ease with transparent mask	339 (33.90)
Less at ease with transparent mask	201 (20.10)
No difference	422 (42.20)
I do not know	38 (3.80)
**Overall, did you prefer the use of one of the masks over the other?**	
Prefer transparent mask	302 (30.20)
Prefer standard mask	267 (26.70)
No preference	394 (39.40)
I do not know	37 (3.70)
**Were you able to tell what emotion the healthcare provider was expressing while wearing the transparent mask?**	
Yes	776 (77.60)
No	143 (14.30)
I do not know	81 (8.10)
**Were you able to tell what emotion the healthcare provider was expressing while wearing the standard mask?**	
Yes	216 (21.60)
No	697 (69.70)
I do not know	87 (8.70)
**Many people in the US are now wearing standard masks as a means to prevent the spread of COVID-19, which usually cover their mouth and most of their face. Do you generally find it harder to communicate with people who are wearing standard masks?**	
Harder to communicate when people are wearing standard masks	416 (41.60)

© 2021 Chu JN et al. *JAMA Network Open.*

Equally well, whether they are wearing a standard mask or not	478 (47.80)
I haven't spoken to anyone wearing a mask	24 (2.40)
I do not know	82 (8.20)
**Considering your day to day interactions with people wearing standard masks, do you think you would be able to understand or hear people better who are wearing transparent masks?**	
Easier to communicate with people who are wearing transparent masks	379 (37.90)
Equally well, whether they wore a standard mask or not	440 (44.00)
I haven't spoken to anyone wearing a mask	30 (3.00)
I do not know	151 (15.10)
**Considering your relationship with your own health care provider(s), do you think that your comfort level speaking with them would be different based on what type of mask they were using?**	
I think I would be more comfortable if they were wearing a transparent mask	247 (24.70)
I think I would be more comfortable if they were wearing a standard mask	168 (16.80)
No preference	470 (47.00)
I do not have a healthcare provider	47 (4.70)
I do not know	68 (6.80)
**Would you prefer to see a health care provider who was wearing a transparent mask or one who was wearing a standard mask?**	
A health care provider who wore a transparent mask	280 (28.00)
A health care provider who wore a standard mask	179 (17.90)
No preference	489 (48.90)
I do not know	52 (5.20)
**Which healthcare provider would you trust more to care for you?**	
A healthcare provider who wore a transparent mask	157 (15.70)
A healthcare provider who wore a standard mask	153 (15.30)
No preference	629 (62.90)
I do not know	61 (6.10)
**How do you feel about wearing masks as a public health response to COVID-19?**	
I agree with wearing masks as a public health measure	701 (70.10)
I disagree with wearing masks as a public health measure	137 (13.70)
I neither agree or disagree with wearing masks as a public health measure	112 (11.20)
I do not know	50 (5.00)
**Do you wear a mask when out in public?**	
Yes, I wear mask	774 (77.40)
No, I do not wear a mask	53 (5.30)
Yes, I sometimes wear a mask	173 (17.30)
**Due to the widespread use of face masks as a result of the public health response to COVID-19, have you experienced any difficulty communicating with others who are wearing face masks?**	
Yes	518 (51.80)
No	482 (48.20)
**If yes to question 16, how would you rate the level of difficulty?**	
Low, some difficulty	221 (22.10)
Moderate, considerable difficulty	203 (20.30)
High, difficult	71 (7.10)
Not difficult	19 (1.90)
Other	4 (0.40)
Did not select yes to question 16	482 (48.20)
**What challenges have the use of face masks created for you when communicating with others? Please select all that apply.**	
It did not create any challenges for me	222 (22.20)
It prevented my ability to see others lips/mouth in order to read lips	296 (29.60)

© 2021 Chu JN et al. *JAMA Network Open.*

It muffled the other person's voice and reduced sound clarity	579 (57.90)
It reduced the volume or sound of the other person's voice	437 (43.70)
Unsure	56 (5.60)
Other	40 (4.00)
**What communication challenges are you concerned about regarding the use of face masks? Please select all that apply.**	
None- I am not concerned about any challenges regarding face masks	261 (26.10)
Inability to see other people's lips/mouth in order to lipread	235 (23.50)
Muffling of the people's voice and reduced sound clarity	529 (52.90)
Reduced volume or sound of the other person's voice	410 (41.00)
Misunderstanding others or not catching what they said	420 (42.00)
Unsure	48 (4.80)
Other	19 (1.90)
**In the last month, how often have you used a mask with a transparent window?**	
Every day	62 (6.20)
Once a week or more	42 (4.20)
Less than once a week and more than once a month	40 (4.00)
Not at all	856 (85.60)
**How well did the transparent mask meet your communication needs?**	
Very well	48 (4.80)
Somewhat well	39 (3.90)
Neutral	36 (3.60)
Not very well	7 (0.70)
Not at all	3 (0.30)
Not applicable	867 (86.70)
**Would you prefer a mask with:**	
Ear loops	580 (58.00)
Strings that tie behind the head	100 (10.00)
Elastic straps that go over the head	99 (9.90)
No preference	179 (17.90)
Other	42 (4.20)
**Would you find any of the following features helpful if they were incorporated into your or others' masks? Please select all that apply.**	
Bluetooth speaker for voice amplification or recognition	176 (17.60)
A light feature to illuminate the room around you (e.g., in a dark room)	89 (8.90)
A light to illuminate the mouth and lips	54 (5.40)
A light indicator that turns on when you or someone else is communicating	85 (8.50)
A sensor that monitors vital signs (e.g., heart rate, temperature, respiratory rate)	103 (10.30)
A sensor to monitor the external environment (e.g., air quality or infection risk)	177 (17.70)
A sensor to provide feedback on mask fit (lights or feedback on quality of seal)	129 (12.90)
Other	18 (1.80)
**Could you see yourself wearing a mask that incorporated advanced technologies?**	
Yes, I could see myself wearing a mask that incorporated advanced technologies	261 (26.10)
No, I could not see myself using a mask that incorporated advanced technologies	458 (45.80)
Unsure	143 (14.30)
No preference	138 (13.80)
**n (Total) = 1000**	

© 2021 Chu JN et al. *JAMA Network Open.*

eTable 3. General Health Care Worker Demographic Characteristics

Survey Question	n (%)
**What is your age?**	
18-24 years old	5 (4.07)
25-34 years old	41 (33.33)
35-44 years old	32 (26.02)
45-54 years old	22 (17.89)
55-64 years old	16 (13.01)
65-74 years old	5 (4.07)
Over 75 years old	2 (1.63)
**What is your gender?**	
Male	39 (31.71)
Female	84 (68.29)
Non-Binary/Third Gender	0 (0.00)
I prefer not to answer	0 (0.00)
**Are you of Hispanic or Latino origin?**	
Yes	3 (2.44)
No	119 (96.75)
Unanswered	1 (0.81)
**Which of the following best describes your racial background?**	
From multiple races	2 (1.63)
Black or African-American	3 (2.44)
Asian	17 (13.82)
Native Hawaiian or Other Pacific Islander	0 (0.00)
White or Caucasian	90 (73.17)
South Asian	5 (4.07)
Prefer not to answer	3 (2.44)
Other/Prefer to self-describe	3 (2.44)
**What is the highest degree or level of school you have completed?**	
High school degree or equivalent	1 (0.81)
Some college but no degree	1 (0.81)
Associate's Degree	6 (4.88)
Bachelor's Degree	32 (26.02)
Master's Degree	18 (14.63)
Professional Degree	52 (42.28)
Doctorate	11 (8.94)
Unanswered	2 (1.63)
**Which of the following best describes your employment status?**	
Employed full-time	110 (89.43)
Employed part-time	10 (8.13)
Not employed, looking for work	0 (0.00)
Not employed, NOT looking for work	0 (0.00)
Disabled, not able to work	0 (0.00)
Retired	1 (0.81)
Other	1 (0.81)
Unanswered	1 (0.81)
**What is your household income?**	
$10,000-$50,000	9 (7.32)
$50,000-$75,000	15 (12.20)
$75,000-$100,000	12 (9.76)

© 2021 Chu JN et al. *JAMA Network Open.*

$100,000-$150,000	16 (13.01)
Greater than $150,000	68 (55.28)
Unanswered	3 (2.44)
**n (Total) = 123**	

© 2021 Chu JN et al. *JAMA Network Open.*

eTable 4. Questions Regarding Health Care Occupation and Mask Use at Work

	n (%)		
**Survey Question**	**General HCWs**	**DHH HCWs**	**Total**
**What type of job do you currently have in the healthcare field?**			
Nurse	25 (20.33)	10 (22.22)	35 (20.83)
Medical technician	1 (0.81)	0 (0.00)	1 (0.60)
Healthcare administration	7 (5.69)	0 (0.00)	7 (4.17)
Attending physician	42 (34.15)	5 (11.11)	47 (27.98)
Fellow/resident	20 (16.26)	4 (8.89)	24 (14.29)
Healthcare student	N/A	4 (8.89)	4 (2.38)
Advanced Practice Provider	15 (12.2)	5 (11.11)	20 (11.90)
Sign Language Interpreter	N/A	4 (8.89)	4 (2.38)
Other	13 (10.57)	13 (28.89)	26 (15.47)
**Have you been working with or taking care of suspected COVID-19 patients?**			
Yes	88 (71.54)	19 (42.22)	107 (63.69)
No	35 (28.46)	26 (57.78)	61 (36.31)
**Are you currently wearing a mask to see patients?**			
Yes, all of the time	119 (96.75)	39 (86.67)	158 (94.04)
No, never	2 (1.63)	3 (6.67)	5 (2.98)
Sometimes, but not always	2 (1.63)	3 (6.67)	5 (2.98)
**Have you found that wearing a mask at work has made it more difficult for you to communicate with patients?**			
Yes	88 (71.54)	39 (86.67)	127 (75.60)
No	32 (26.02)	4 (8.89)	36 (21.43)
Not applicable	3 (2.44)	2 (4.44)	5 (2.97)
**Population Total (n)**	**n=123**	**n=45**	**n=168**

© 2021 Chu JN et al. *JAMA Network Open.*

eTable 5. General Health Care Worker Survey Questions

Survey Question	n (%)
**Considering the video you have just watched (video #1), how was the healthcare provider feeling?**	
Happy	109 (88.62)
Sad	1 (0.81)
I do not know	13 (10.57)
**Consider the video that you have just watched (video #1) demonstrating the use of a transparent mask. As a healthcare provider, how would you feel wearing a transparent mask to interact with patients?**	76 (61.79)
Positively	5 (4.07)
Negatively	38 (30.89)
Neutral	4 (3.25)
I do not know	
**Considering the video you have just watched (video #2), how was the healthcare provider feeling?**	
Happy	25 (20.49)
Sad	1 (0.82)
I do not know	96 (78.69)
**Consider the video you just watched (video #2) demonstrating the use of a standard mask. As a healthcare provider, how would you feel wearing a standard mask to interact with patients?**	20 (16.26)
Positively	28 (22.76)
Negatively	74 (60.16)
Neutral	1 (0.81)
I do not know	
**Did you feel that communication was clearer in the video of the transparent mask or in the video of the standard mask?**	106 (86.18)
Communication was clearer with the transparent mask	1 (0.81)
Communication was clearer with the standard mask	12 (9.76)
No difference	4 (3.25)
I do not know	
**Overall, did you prefer the use of one of the masks over the other?**	65 (52.85)
Prefer transparent mask	10 (8.13)
Prefer standard mask	31 (25.20)
No preference	17 (13.82)
I do not know	
**Widespread use of masks in healthcare settings is now customary due to COVID-19, requiring healthcare providers to wear masks while interacting with patients. Do you generally find it harder to communicate with patients while wearing a standard mask?**	84 (68.85)
It is harder to communicate with patients while wearing a standard mask	26 (21.31) / 12 (9.84)
I can communicate with patients equally well	
I do not know	
**Consider your daily interactions with patients who are wearing standard masks. Do you think you would be able**	

© 2021 Chu JN et al. *JAMA Network Open.*

**to understand or hear them better if they wore a transparent mask?**<br>*It is easier to communicate with patients who are wearing transparent masks*<br>*I can understand patients equally well*<br>*I haven't spoken to any patients wearing a mask*<br>*I do not know*	75 (60.98)<br>26 (21.14)<br>2 (1.63)<br>20 (16.26)
**Do you feel that you would be able to communicate more effectively/convey empathy better with patients if you were wearing a transparent mask?**<br>*I would be able to communicate better while wearing transparent mask*<br>*I can communicate equally well*<br>*I do not know*	95 (77.24)<br>22 (17.89)<br>6 (4.88)
**Do you think patients would feel more or less at ease if the healthcare provider was wearing a transparent mask?**<br>*Patients would be more at ease if healthcare providers wore a transparent mask*<br>*Patients would be less at ease if healthcare providers wore a transparent mask*<br>*No difference*<br>*I do not know*	75 (60.98)<br>6 (4.88)<br>17 (13.82)<br>25 (20.33)
**Due to the widespread use of face masks as a result of the public health response to COVID-19, have you experienced any difficulty communicating with patients who are wearing face masks?**<br>*Yes*<br>*No*	92 (74.80)<br>31 (25.20)
**If yes to question 11, how would you rate the level of difficulty?**<br>*Low, some difficulty*<br>*Moderate, considerable difficulty*<br>*High, difficult*<br>*Did not select yes to question 11*<br>*Unanswered*	60 (48.78)<br>27 (21.95)<br>4 (3.25)<br>31 (24.20)<br>1 (0.82)
**What challenges did the use of face masks create for you when communicating with patients? Please select all that apply.**<br>*It did not create any challenges for me*<br>*It prevented my ability to see others lips/mouth in order to read lips*<br>*It muffled the other person's voice and reduced sound clarity*<br>*It reduced the volume or sound of the other person's voice*<br>*I do not know*<br>*Other*	14 (11.38)<br>54 (43.90)<br>97 (78.86)<br>86 (69.92)<br>1 (0.81)<br>8 (6.50)
**What communication challenges are you concerned about regarding the use of face masks? Please select all that apply.**<br>*None- I am not concerned about any challenges regarding face masks*<br>*Inability to see other people's lips/mouth in order to lipread*<br>*Muffling of the people's voice and reduced sound clarity*<br>*Reduced volume or sound of the other person's voice*	13 (10.57)<br>51 (41.46)<br>87 (70.73)<br>82 (66.67)<br>87 (70.73)<br>3 (2.44)<br>6 (4.88)

© 2021 Chu JN et al. *JAMA Network Open.*

Misunderstanding others or not catching what they said	
I do not know	
Other	
**In the last month, how often have you used a mask with a transparent window?**	
Every day	3 (2.44)
Less than once a week and more than once a month	1 (0.81)
Not at all	119 (96.75)
**Would you prefer a mask with:**	
Ear loops	72 (58.54)
Strings that tie behind the head	9 (7.32)
Elastic straps that go over the head	27 (21.95)
No preference	14 (11.38)
Other	1 (0.81)
**Would you find any of the following features helpful if they were incorporated into your or others' masks? Please select all that apply.**	40 (32.52)
Bluetooth speaker for voice amplification or recognition	15 (12.20)
A light feature to illuminate the room around you	13 (10.57)
A light to illuminate the mouth and lips	24 (19.51)
A light indicator that turns on when you or someone else is	18 (14.63)
communicating	37 (30.08)
A sensor that monitors vital signs	68 (55.28)
A sensor to monitor the external environment	4 (3.25)
A sensor to provide feedback on the fit masks	
Other	
**Could you see yourself wearing a mask that incorporated advanced technologies?**	
Yes, I could see myself wearing a mask that incorporated	59 (47.97)
advanced technologies	20 (16.26)
No, I could not see myself using a mask that incorporated	41 (33.33)
advanced technologies	3 (2.44)
I do not know	
Other	
**n (Total) = 123**	

© 2021 Chu JN et al. *JAMA Network Open.*

eTable 6. Demographic Characteristics for DHH Health Care Workers

Survey Question	n (%)
**Age**	
18-24 years old	1 (2.22)
25-34 years old	10 (22.22)
35-44 years old	7 (15.56)
45-54 years old	11 (24.44)
55-64 years old	12 (26.67)
65-74 years old	14 (8.89)
Over 75 years old	0 (0.00)
**Gender**	
Male	15 (33.33)
Female	30 (66.67)
Non-Binary/Third Gender	0 (0.00)
I prefer not to answer	0 (0.00)
Other- please specify	0 (0.00)
**Are you of Hispanic or**	
**Latino origin?**	2 (4.44)
Yes	43 (95.56)
No	
**Which of the following best**	
**describes your racial**	
**background?**	0 (0.00)
American Indian or	1 (2.22)
Alaskan Native	1 (2.22)
Black or African	0 (0.00)
American	41 (91.11)
Asian	2 (4.44)
Native Hawaiian or	0 (0.00)
Pacific Islander	0 (0.00)
White or Caucasian	0 (0.00)
South Asian	
From multiple races	
I prefer not to answer	
Other/Prefer to self-	
describe	
**What is the highest degree**	
**or level of school you have**	
**completed?**	0 (0.00)
Less than a high school	1 (2.22)
diploma	0 (0.00)
High school degree or	0 (0.00)
equivalent	9 (20.00)
Some college but no	14 (31.11)
degree	16 (35.56)
Associate's Degree	5 (11.11)
Bachelor's Degree	
Master's Degree	
Professional Degree	
Doctorate	
**Which of the following best**	
**describes your employment**	
**status?**	27 (60.00)
Employed full-time	10 (22.22)

© 2021 Chu JN et al. *JAMA Network Open.*

Employed part-time<br>*Not employed, looking for work*<br>*Not employed, NOT looking for work*<br>*Retired*<br>*Disabled, not able to work*<br>*Other- please specify*	1 (2.22)<br>2 (4.44)<br>1 (2.22)<br>1 (2.22)<br>3 (6.67)
**What is your household income?**<br>*Below $10,000.0*<br>*$10,000-$50,000.0*<br>*$50,000-$75,000.0*<br>*$75,000-$100,000.0*<br>*$100,000-$150,000.0*<br>*Greater than $150,000.0*	 1 (2.22)<br>5 (11.11)<br>11 (24.44)<br>6 (13.33)<br>9 (20.00)<br>13 (28.89)
**n (Total) = 45**	

© 2021 Chu JN et al. *JAMA Network Open.*

eTable 7. Questions Related to Hearing Loss

Survey Question	n (%)
**How would you categorize your degree of hearing loss?**	
Normal loss	1 (2.22)
Slight hearing loss	0 (0.00)
Mild hearing loss	1 (2.22)
Moderate hearing loss	5 (11.11)
Moderately severe hearing loss	9 (20.00)
Severe hearing loss	6 (13.33)
Profound hearing loss	16 (35.56)
Other	7 (15.56)
**What is your preferred method of communication? Please**	
**select all that apply.**	35 (83.33)
Auditory input/Listening	31 (68.89)
Lip-reading	9 (20.00)
Sign language	15 (33.33)
Written notes or reading text	3 (6.67)
Other	
**Do you use an assistive listening device, such as a hearing aid, for hearing assistance?**	
Yes	38 (84.44)
No	7 (15.56)
**If yes to question 3, what type of assistive listening device do you use for hearing assistance?**	
Hearing Aid	21 (46.67)
Cochlear Implant	14 (31.11)
Bone Conduction Implant System	1 (2.22)
Other	3 (6.67)
Not applicable	6 (13.33)
**If you utilize a medical device for hearing assistance, do you experience any mask-fit interference with your**	
**device?**	27 (60.00)
Yes, my mask interferes with my hearing device	0 (0.00)
Yes, I cannot use a mask because it interferes with my	9 (20.00)
hearing device	0 (0.00)
No, my mask does not interfere	2 (4.44)
I do not know	7 (15.56)
Other	
Not applicable	
**Have you ever failed a N95 fit-test due to the strap placement of the mask interfering with an assistive**	
**listening device, such as a cochlear implant?**	1 (2.22)
Yes, I have failed an N95 fit-test	20 (44.44)
No, I have never failed	24 (53.33)
Not applicable	
**How do you feel about the use of face masks regarding their potential effect on communication? Please select all**	
**that apply.**	4 (8.89)
I am not worried about the impact of facemasks on my	32 (71.11)
communication	32 (71.11)
It will make living with a hearing loss much more difficult	17 (37.78)
I am anxious or stressed about their effect on my ability to	20 (44.44)
communicate	39 (86.67)
I am worried that it will isolate me	1 (2.22)

© 2021 Chu JN et al. *JAMA Network Open.*

I am worried that it will reduce my independence	
I am worried I will more frequently misunderstand people	
Other	
**Since the COVID 19 Outbreak has made standard masks, like N95s, a requirement for HCWs at all times, how has this impacted your work?**	
It has not impacted my work	25 (55.56)
I have been relocated to another ward	2 (4.44)
I have been displaced	2 (4.44)
My hours have been reduced at work	1 (2.22)
Other	15 (33.33)
**Could you see yourself wearing a mask that incorporated advanced technologies?**	
Yes, I could see myself wearing a mask that incorporated	31 (68.89)
advanced technologies	5 (11.11)
No, I could not see myself using a mask that incorporated	7 (15.56)
advanced technologies	1 (2.22)
I do not know	1 (2.22)
No preference	
Other	
**Due to the widespread use of face masks as a result of the public health response to COVID-19, have you experienced any difficulty communicating with patients**	
**who are wearing face masks?**	44 (97.78)
Yes	1 (2.22)
No	
**If yes to the previous question, how would you rate the**	
**level of difficulty?**	6 (13.33)
Low, some difficulty	18 (40.00)
Moderate, considerable difficulty	18 (40.00)
High, difficult	2 (4.44)
Other	1 (2.22)
Not applicable	
**What challenges did the use of face masks create for you when communicating with patients? Please select all that**	
**apply.**	1 (2.22)
It did not create any challenges for me	41 (91.11)
It prevented my ability to see the patient's lips/mouth in	40 (88.89)
order to read lips	29 (64.44)
It muffled the patient's voice and reduced sound clarity	1 (2.22)
It reduced the volume or sound of the patient's voice	2 (4.44)
I do not know	
Other	
**What communication challenges are you concerned about regarding the use of face masks? Please select all that**	
**apply.**	1 (2.22)
None	40 (88.89)
Inability to see the patient's lips/mouth in order to lipread	38 (84.44)
Muffling of the patient's voice and reduced sound clarity	32 (71.11)
Reduced volume or sound of the patient's voice	35 (77.78)
Misunderstanding my patients or not catching what they	0 (0.00)
said	1 (2.22)
I do not know	
Other	

© 2021 Chu JN et al. *JAMA Network Open.*

**How do you feel about the use of face masks regarding**	
**their potential effect on communication? Please select all**	
**that apply.**	4 (8.89)
I am not worried about the impact of facemasks on my	32 (71.11)
communication	32 (71.11)
It will make living with a hearing loss much more difficult	17 (37.78)
I am anxious or stressed about their effect on my ability to	20 (44.44)
communicate	39 (86.67)
I am worried that it will isolate me	1 (2.22)
I am worried that it will reduce my independence	
I am worried I will more frequently misunderstand people	
Other	
**n (Total) = 45**	

© 2021 Chu JN et al. *JAMA Network Open.*

eTable 8. DHH Health Care Worker Survey Questions

Survey Question	n (%)
**Considering the video you have just watched (video #1), how was the healthcare provider feeling?**	
Happy	38 (84.44)
Sad	0 (0.00)
I do not know	7 (15.56)
**Consider the video that you have just watched (video #1) demonstrating the use of a transparent mask. As a healthcare provider, how would you feel wearing a transparent mask to interact with patients?**	
Positively	37 (82.22)
Negatively	1 (2.22)
Neutral	4 (8.89)
I do not know	3 (6.67)
**Considering the video you have just watched (video #2), how was the healthcare provider feeling?**	
Happy	11 (24.44)
Sad	2 (4.44)
I do not know	32 (71.11)
**Consider the video you just watched (video #2) demonstrating the use of a standard mask. As a healthcare provider, how would you feel wearing a standard mask to interact with patients?**	
Positively	0 (0.00)
Negatively	29 (64.44)
Neutral	13 (28.89)
I do not know	3 (6.67)
**Did you feel that communication was clearer in the video of the transparent mask or in the video of the standard mask?**	
Communication was clearer with the transparent mask	44 (97.78)
Communication was clearer with the standard mask	0 (0.00)
No difference	1 (2.22)
I do not know	0 (0.00)
**Overall, did you prefer the use of one of the masks over the other?**	
Prefer transparent mask	41 (91.11)
Prefer standard mask	0 (0.00)
No preference	3 (6.67)
I do not know	1 (2.22)
**Widespread use of masks in healthcare settings is now customary due to COVID-19, requiring healthcare providers to wear masks while interacting with patients. Do you generally find it harder to communicate with patients while wearing a standard mask?**	
It is harder to communicate with patients while wearing a standard mask	39 (86.67)
I can understand patients equally well	1 (2.22)
I haven't spoken to any patients wearing a mask	5 (11.11)
I do not know	0 (0.00)
**Consider your daily interactions with patients who are wearing standard masks. Do you think you would be able to understand or hear them better if they wore a transparent mask?**	
It is easier to communicate with patients who are wearing transparent masks	41 (91.11)
I can understand patients equally well	1 (2.22)
I do not know	3 (6.67)
**Do you feel that you would be able to communicate more effectively/convey empathy better with patients if you were wearing a transparent mask?**	
I would be able to communicate better while wearing transparent mask	41 (91.11)

© 2021 Chu JN et al. *JAMA Network Open.*

I can communicate equally well	2 (4.44)
I do not know	2 (4.44)
**Did you think patients would feel more or less at ease if the healthcare provider was wearing a transparent mask?**	
Patients would be more at ease if healthcare providers wore a transparent mask	34 (75.56)
Patients would be less at ease if healthcare providers wore a transparent mask	2 (4.44)
No difference	0 (0.00)
I do not know	9 (20.00)
**Do you think you would feel more at ease communicating with patients who were wearing a transparent mask?**	
I would feel more at ease with patients who wore a transparent mask	39 (86.67)
I would feel less at ease with patients who wore a transparent mask	1 (2.22)
No difference	3 (6.67)
I do not know	2 (4.44)
**In the last month, how often have you used a mask with a transparent window?**	
Every day	5 (11.11)
Once a week or more	8 (17.78)
Less than once a week and more than once a month	7 (15.56)
Not at all	25 (55.56)
**How well did the transparent mask meet your communication needs?**	
Very well	6 (13.33)
Somewhat well	10 (22.22)
Neutral	3 (6.67)
Not very well	3 (6.67)
Not at all	0 (0.00)
Not applicable	23 (51.11)
**How much do existing mask designs interfere with your use of hearing assistance devices?**	
Frequently	14 (31.11)
Sometimes	18 (40.00)
Not at all	6 (13.33)
Not applicable	7 (15.56)
**Would you prefer a mask with:**	
Ear loops	12 (26.67)
Strings that tie behind the head	10 (22.22)
Elastic straps that go over the head	15 (33.33)
No preference	7 (15.56)
Other-please specify	1 (2.22)
**Since the COVID 19 Outbreak has made standard masks, like N95s, a requirement for HCWs at all times, how has this impacted your work?**	
It has not impacted my work	25 (55.56)
I have been relocated to another ward	2 (4.44)
I have been displaced	2 (4.44)
My hours have been reduced at work	1 (2.22)
Other- please specify	15 (33.33)
**n (Total) = 45**	

© 2021 Chu JN et al. *JAMA Network Open.*

eTable 9. Response to Videos of a Study Author Wearing a Transparent N95 Mask and Standard Opaque N95 Mask

	n %							
	**General Population**		**General HCWs**		**DHH HCWs**		**Total**	
	Transparent Mask	*Standard Mask*	*Transparent Mask*	*Standard Mask*	*Transparent Mask*	*Standard Mask*	*Transparent Mask*	*Standard Mask*
**Considering the video you have just watched, how was the healthcare worker feeling?**								
Happy	781(78.10)	201 (20.10)	109 (88.62)	25 (20.49)	38 (84.44)	11 (24.44)	928(79.45)	237 (20.31)
Sad	53 (5.30)	94 (9.40)	1 (0.81)	1 (0.81)	0 (0.00)	2 (4.44)	54 (4.62)	97 (8.31)
I do not know	166 (16.60)	705 (70.50)	13 (10.57)	96 (78.69)	7 (15.56)	32 (71.11)	186(15.92)	833 (71.38)
	P<0.001		P<0.001		P<0.001		P<0.001	
**Consider the video that you have just watched of the use of a transparent mask. How would you feel if a healthcare provider you are interacting with wore a transparent N95 mask/standard opaque N95 mask?/As a healthcare provider, how would you feel wearing a transparent N95 mask/standard opaque N95 mask to interact with patients?**								
Positively	450 (45.00)	308 (30.80)	76 (61.79)	20 (16.26)	37 (82.22)	0 (0.00)	563 (48.20)	328 (28.08)
Negatively	118 (11.80)	82 (8.20)	5 (4.07)	28 (22.76)	1 (2.22)	29 (64.44)	124 (10.62)	139 (11.90)
Neutral	366 (36.60)	540 (54.00)	38 (30.89)	74 (60.16)	4 (8.89)	13 (28.89)	408 (34.93)	627 (53.68)
I do not know	66 (6.60)	70 (7.00)	4 (3.25)	1 (0.81)	3 (6.67)	3 (6.67)	73 (6.25)	74 (6.34)
**p-value (<0.05)**	P<0.01		P<0.01		P=<0.01		P<0.01	
**Population Total (n)**	**n=1000**		**n=123**		**n=45**		**n=1168**	

© 2021 Chu JN et al. *JAMA Network Open.*

eMethods.

To accomplish this, three study team members (JC, TC, JNC) first read all open-ended responses and generated key themes based on an initial reading. Next, the open-ended responses were read by participants and categorized into these themes. Any additional themes that emerged in the second reading were noted and shared with the study team. Finally, all key themes and responses in the framework matrix were shared and discussed with the entire team who came to a consensus on key concepts that emerged from open ended responses.

© 2021 Chu JN et al. *JAMA Network Open.*

eTable 10. Subset of Respondents' Comments from Each Population on Challenges and Concerns Regarding the Widespread Use of Facemasks

**General Population**
People cannot hear me.
Eyeglasses fogging up (n=8)
It's hard to breathe in mask.
I think it reduces all communication, smiles, friendly gestures and it's extremely sad.
It disguised facial cues/clues about conversation context/emotion.
**General HCWs**
It's uncomfortable and itchy. My consultation with patients is shorter because I wanted to take the mask off.
The patient could not read my lips.
My own voice is muffled, takes longer to speak loud and clear.
Limited conveyance of emotion / facial expressions.
More difficulty interpreting patients' mood or state of mind.
**DHH HCWs**
Challenges
There are some voice pitches I just cannot hear.
Please understand that I am doing tele-mental health and in doing so I can see client's lips, so I answered these questions when I am interacting with other people who wear masks.
Concerns
It's also a huge hassle to say, to everyone when you can't hear that I wear hearing aids and can't hear what you are saying. Even when the people already know.
Being unable to work as a nurse due to safety reasons with severe hearing loss.

© 2021 Chu JN et al. *JAMA Network Open.*

eTable 11. Mask Preference and Type

	n (%)				
	**General Population**	**General HCWs**	**DHH HCWs**	**Total**	***p***
**In the last month, how often have you used a mask with a transparent window?**					p< 0.001
Every day	62 (6.20)	5 (11.11)	3 (2.44)	70 (5.99)	
Once a week or more	42 (4.20)	8 (17.78)	N/A	50 (4.28)	
Less than once a week and more than once a month	40 (4.00)	7 (15.56)	1 (0.81)	48 (4.11)	
Not at all	856 (85.60)	25 (55.56)	119 (95.76)	1000 (85.62)	
**Would you prefer a mask with:**					p< 0.001
Ear loops	580 (58.00)	72 (58.54)	12 (26.67)	664 (56.85)	
Strings that tie behind the head	100 (10.00)	9 (7.32)	10 (22.22)	119 (10.19)	
Elastic straps that go over the head	99 (9.90)	27 (21.95)	15 (33.33)	141 (12.07)	
No preference	179 (17.90)	14 (11.38)	7 (15.56)	200 (17.12)	
Other	42 (4.20)	1 (0.81)	1 (2.22)	44 (3.77)	
**Would you find any of the following features helpful if they were incorporated into your or others' masks? Please select all that apply.**					N/A
Bluetooth speaker for voice amp/recognition	176 (17.60)	40 (32.52)	24 (53.33)	240 (20.55)	
A light feature to illuminate the room	89 (8.90)	15 (12.20)	10 (22.22)	114 (9.76)	
A light to illuminate mouth/lips	54 (5.40)	13 (10.57)	18 (40.00)	85 (7.28)	
A light indicator that turns on when someone is communicating	85 (8.50)	24 (19.51)	16 (35.56)	125 (10.70)	
A microphone that transmits info to hearing assistance device	N/A	N/A	30 (66.67)	30 (66.67)	
A sensor that monitors vital signs	103 (10.30)	18 (14.63)	19 (42.22)	140 (11.99)	
A sensor to monitor the external environment	177 (17.70)	37 (30.08)	12 (26.67)	226 (19.35)	
A sensor to provide feedback on the fit of masks	129 (12.90)	68 (55.28)	12 (26.67)	209 (17.89)	
Other	18 (1.80)	4 (3.25)	8 (17.78)	30 (2.57)	
**Could you see yourself wearing a mask that incorporated advanced technologies (e.g., Bluetooth for voice amplification/recognition or a light feature to illuminate a room or a person's lips)?**					p< 0.001
Yes, I could see myself wearing a mask that incorporated adv. tech	261 (26.10)	59 (47.97)	31 (68.89)	351 (30.05)	
No, I could not see myself wearing a mask that incorporated adv. tech	458 (45.80)	20 (16.26)	5 (11.11)	483 (41.35)	
I do not know	143 (14.30)	41 (33.33)	7 (15.56)	191 (16.35)	
No preference	138 (13.80)	N/A	1 (2.22)	139 (11.90)	
Other	N/A	3 (2.44)	1 (2.22)	4 (0.34)	
**Population Total (n)**	**n=1000**	**n=123**	**n=45**	**n=1168**	

**p-value < 0.05 was considered statistically significant*
***Questions that were not asked of specific groups were labelled N/A*

© 2021 Chu JN et al. *JAMA Network Open.*
